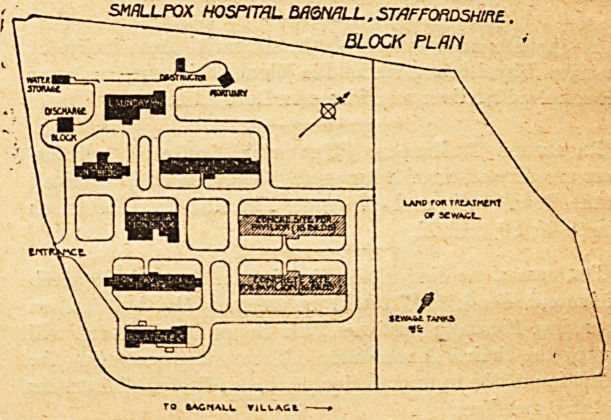# The North Staffordshire Joint Hospital for Small-Pox, at Bagnall

**Published:** 1907-03-16

**Authors:** 


### HOSPITAL ADMINISTRATION.

### CONSTRUCTION AND ECONOMICS.

### THE NORTH STAFFORDSHIRE JOINT HOSPITAL FOR SMALL-POX, AT BAGNALL.

About seven years since the Local Government Board prohibited the reception of small-pox cases at the old hospital at Bucknall, as that institution, although originally intended as a small-pox hospital, was being used for the treatment of other infectious diseases. The local authorities had therefore to look out for a site; not a very easy thing to find, as the hospital must not be within a quarter of a mile of any other hospital, workhouse, or asylum, nor must there be a population of more than 200 within the quarter-mile radius, nor more than 600 within a half-mile radius. Further, there must be a good supply of water; and in addition to all these it has to be borne in mind that landowners often object to sell land for such a purpose, as they naturally fear the depreciation in value of the adjoining land. The Joint Board were fortunately able to surmount all the difficulties, and they obtained an excellent site of 7½ acres, lying between the villages of Bagnall and Milton.

The entrance gates are on the road leading to Milton, and almost opposite these gates on entering the grounds is the administrative block. This is a three-story block, the ground plan of which is a parallelogram, divided up the centre by a corridor having at each end an entrance porch. On the left hand of the main porch are the medical officer's sitting-room and bedroom, the dispensary, the nurses' mess-room, the scullery, and the kitchen; and on the right hand are the matron's sitting-room, the store-room, bath-room and closet, the pantry, and the larder. The first floor contains five bedrooms and the second floor six bedrooms, all being intended for the staff. The general arrangement of this block is decidedly good. South-west of this block is an iron pavilion for twelve beds; and further off in the same direction is the isolation pavilion for six beds, three for each sex. There are two single-bedded wards, which, it may be remarked, the architect has attended to and obtained efficient cross-ventilation. Each division of this pavilion is provided with its own verandah, and the sanitary annexes are thrown out from the verandah. Baths, apparently moveable, are provided.

Eastward of these pavilions are two concrete sites, on which two other pavilions for sixteen beds each could be at any time erected if necessary.

To the north is an iron pavilion for ten beds, and in line with it is another for seven beds. Not far from the latter is the discharging block; and further north is the laundry, with which are incorporated the disinfecting chamber; the ambulance house and the stables, and close to it are the mortuary and the refuse-destructor.

There is nothing specially remarkable about the iron pavilions, but they are quite suitable for the treatment of small-pox cases. The twenty-bedded pavilion is divided into two equal parts by the nurses' duty-room and nurses'

ADMINISTRATION BLOCK·

GROUND FLOOR PLAN

FIRST FLOOR PLAN

2ND FL. CONTAINS 6 BED RMS

bedroom, between which is the passage connecting the two dormitories. Each bed has a window on both sides. The sanitary annexes are placed at the ends of the dormitories; and, although they are not cut off from the wards so effectually as they would be in a modern permanent hospital, there is cross-ventilation, obtainable through the places containing the hot water supply and the sink-room.

Each patient is allowed 2,000 cubic feet of air space. This is by no means a large supply for small-pox patients, but if the renewal of air be constantly attended to, it may perhaps be sufficient.

The drainage has been carefully constructed, the rain-water system being kept quite distinct from the sewage. The latter is conveyed in open channels, and by this arrangement it is claimed that no objectionable matter can find a lodgment. The sewage is first of all passed into a grit and screening chamber, whence it flows over alumine-ferric; then through a "baffling race," to ensure thorough admixture of sewage and chemicals before it is passed into the settling-tanks, which are each of 1,500 gallons capacity. From these tanks the effluent is passed on to the distributing chambers, eventually flowing on to and irrigating the land.

The whole of the permanent buildings is constructed of red brick, having stone sills and tiled roofs.

The architect was Mr. E. Jones, of Hanley; and the contractor for the buildings and the concrete sites was Mr. James Moss, of Milton. The sewage works were carried out by Mr. Bagnall, of Fenton; but the valves and sluices were supplied by Messrs. Adams, of York. The baths, etc., were by Messrs. Winkle, of Stoke.

The cost of the buildings, including boundary walls, drainage, and sewage works, was £7,050. The site cost

£400, and the law charges amounted to £200—just half the cost of the site! £50 had to be given to an adjoining tenant, making the total of £7,700. As the hospital contains twenty-nine beds, the cost per bed would be nearly £200; but then this sum does not, apparently, include the three iron pavilions.

## Figures and Tables

**Figure f1:**
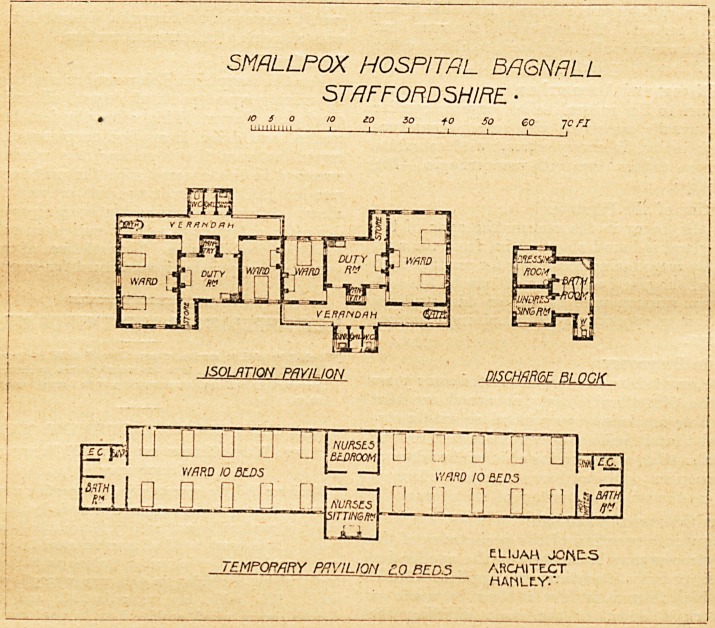


**Figure f2:**
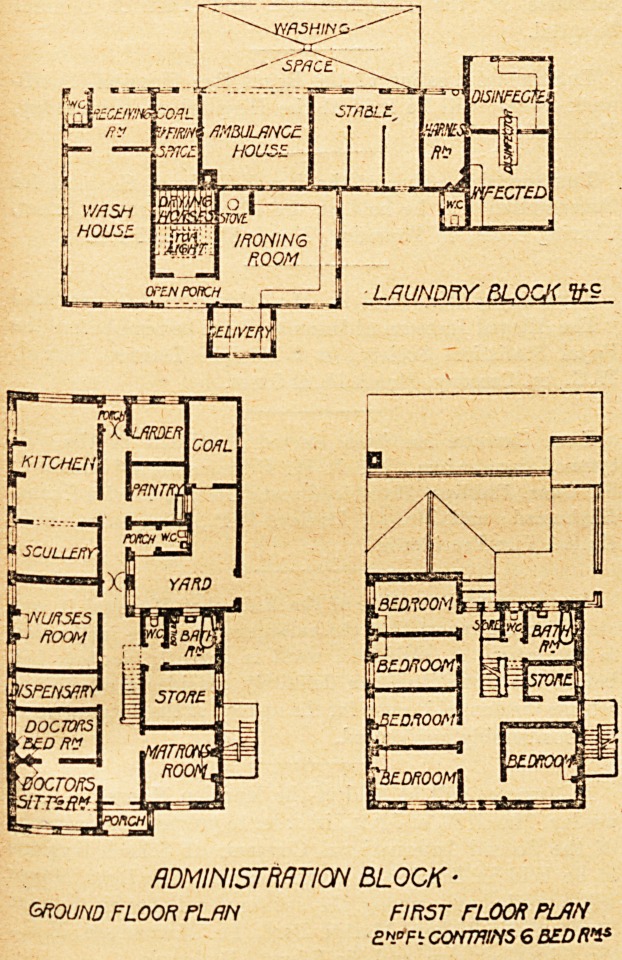


**Figure f3:**